# A Nanoindentation Approach for Time-Dependent Evaluation of Surface Free Energy in Micro- and Nano-Structured Titanium

**DOI:** 10.3390/ma15010287

**Published:** 2021-12-31

**Authors:** Serena De Santis, Edoardo Rossi, Marco Sebastiani, Simona Sennato, Edoardo Bemporad, Monica Orsini

**Affiliations:** 1 Department of Industrial, Electronic and Mechanical Engineering, Roma Tre University, Via Vito Volterra 62, 00146 Rome, Italy; monica.orsini@uniroma3.it; 2 Engineering Department, Università Degli Studi Roma Tre, Via Della Vasca Navale 79, 00146 Rome, Italy; edoardo.rossi@uniroma3.it (E.R.); marco.sebastiani@uniroma3.it (M.S.); edoardo.bemporad@uniroma3.it (E.B.); 3 CNR-ISC Sede Sapienza, Department of Physics, Sapienza University of Rome, Piazzale Aldo Moro 5, 00185 Rome, Italy; simona.sennato@roma1.infn.it

**Keywords:** titanium, surface free energy, nanoindentation, chemical treatment, aging

## Abstract

Surface free energy (SFE) of titanium surfaces plays a significant role in tissue engineering, as it affects the effectiveness and long-term stability of both active coatings and functionalization and the establishment of strong bonds to the newly growing bone. A new contact–mechanics methodology based on high-resolution non-destructive elastic contacting nanoindentation is applied here to study SFE of micro- and nano-structured titanium surfaces, right after their preparation and as a function of exposure to air. The effectiveness of different surface treatments in enhancing SFE is assessed. A time-dependent decay of SFE within a few hours is observed, with kinetics related to the sample preparation. The fast, non-destructive method adopted allowed for SFE measurements in very hydrophilic conditions, establishing a reliable comparison between surfaces with different properties.

## 1. Introduction

Due to their unique mechanical and physicochemical properties, Titanium (Ti) and its alloys are among the most used materials in biomedical engineering for dental and orthopedic applications. They are flexible, low-density materials with high tensile strength, high corrosion resistance in body fluids, and excellent biocompatibility. The characteristic self-healing titanium oxide layer (TiO_2_) spontaneously formed on Ti surfaces is responsible for many of these interesting properties. Unfortunately, the price for the high stability gained is a limited ability to interact with the surrounding environment and to establish a direct bond with the newly growing bone tissue. As a consequence, osseointegration of titanium-based implants is slow and can be compromised by poor biological sealing, which is responsible for loose fixation with bone tissue and postoperative infection. Surface modifications are the key for adding the lacking biofunctionality and further improving Ti biocompatibility while retaining its favorable bulk properties. Many different strategies have been explored to achieve these important goals, such as the addition of bioactive layers by coating techniques [1,2] or the functionalization with suitable polymers and biomolecules [3,4,5]. Chemical etching of titanium surfaces can be considered as both a surface modification for osseointegration improvement [6,7] and as a pre-treatment for the above-mentioned approaches. Treatment of titanium surfaces with acids or alkali modifies surface morphology, producing uniform surface finishing with increased roughness and micrometer and/or nanometer scale patterns. This kind of topographic features was demonstrated to significantly influence cell behaviors, such as cell morphology, migration, adhesion, proliferation, and differentiation [6,7,8]. The effective removal of surface contaminants and the increase in exposed surface area performed by chemical etching is also responsible for an increase in available active site, which is a key requirement to achieve extensive and homogenous surface functionalization with bioactive molecules. Moreover, chemical etching was proven to profoundly alter the surface free energy (SFE), playing a significant role in determining the success of the modified implants. In fact, SFE strongly influences coating adhesion [9] and the kind and extent to which different functionalities can be introduced. It also has a critical effect on the wettability of the final product and hence on its initial conditioning by proteins [10] and subsequent cell response [11]. Therefore, a precise knowledge of the SFE associated with a specific surface treatment constitutes a valid support for the design of synthetic protocols and the development of materials capable of inducing rapid and stable osteointegration. Increasing interest in designing and characterizing materials with high SFE was demonstrated in recent studies [12,13], with attention paid on the role of surface modification [14,15,16,17,18]. Different methods for calculating SFE are known in the literature [19,20]. Despite their differences, most of these methods rely on determining the Young contact angle (θ_y_) between the surface of interest and a liquid drop resting on it, whose spreading is driven by the solid–liquid interactions [21]. Accurately measuring θ_y_ requires flat, rigid, perfectly smooth, and chemically homogeneous surfaces with no chemical reactions with the measuring liquid employed. Surfaces produced by manufacturing procedures are far from ideal and complex data interpretation is required to compensate for the strong influence of surface roughness and chemical heterogeneity on the measured θ [22]. The number and nature of liquids to be used for the measurements are also a matter of discussion: an increasing number of probes is obviously expected to improve the accuracy of the results, but some studies evidenced that the use of several liquids could lead to the incorrect outcome [23]. In addition, measurement of SFE as a function of time after the treatment is usually not possible by contact angle measurements over the same sample.

In this work, in the context of the recent ongoing advances in nanomechanical testing methodologies via commercially available nanoindenters [24,25], we applied a recently proposed contact-mechanics non-destructive methodology for the SFE determination of chemically treated titanium surfaces [26]. This new protocol, based on the elastic contact between a nanoindenter spheroconical tip and the surface under investigation, allows the assessing SFE of materials by measuring the pull-off adhesive forces required to separate the indenter tip from the sample surface in a carefully controlled environment. The SFE of titanium surfaces subjected to four out of the most common chemical treatments was determined right after their preparation. Samples were characterized under protective nitrogen atmosphere, using a well-established nitrogen purging and fluxing methodology to assess precise environmental control (crucial for SFE measurements) [25,26] and then exposed to air to assess time-related changes in SFE, which would have a high impact on the material properties and storage conditions eventually required for their preservation.

## 2. Materials and Methods

### 2.1. Materials

Titanium foils (thickness 0.127 mm, 99.7% trace metals basis), hydrofluoric acid (HF, 48%), hydrogen peroxide (H_2_O_2_, 30%) were purchased from Merk Life Science, Milano, Italy. Hydrochloric acid (HCl, 37%), sodium hydroxide (NaOH, 98%) and sulfuric acid (H_2_SO_4_, 96%) were supplied from Carlo Erba Reagents, Milano, Italy. All reagents were used without further purification.

### 2.2. Samples Preparation

Commercially pure titanium (Ti) foil was cut into 1.5 × 2 cm^2^ pieces. All samples were cleaned by an ultrasonic bath in water/acetone 50:50 and ethanol for 10 min each, rinsed with distilled water and dried under a nitrogen stream at ambient temperature.

Chemical etching was performed soaking samples in the following solution: 2%wt HF solution for 45 s (Ti-HF), 18%wt HCl solution at 80 °C for 10 min (Ti-HCl), 5M NaOH 80 °C for 2 h hours (Ti-NaOH), in H_2_SO_4_/H_2_O_2_ 50:50 (piranha solution) at 80 °C for 30 min (Ti-Pir). After treatments, samples were rinsed with distilled water until the pH value of the mixture becomes neutral and were cleaned in an ultrasonic bath in distilled water for 5 min and then in ethanol for 5 min to remove any debris. They were finally dried under a nitrogen stream at ambient temperature and immediately stored in N_2_ atmosphere.

### 2.3. Scanning Electron Microscopy

Morphological observations of the titanium surfaces were obtained by scanning electron microscopy (SEM) using a Zeiss Gemini SIGMA 300 FEG SEM (Jena, Germany). Micrographs were obtained at 5 kV, with a backscattered detector and working distance of 7.5 mm.

### 2.4. Atomic Force Microscopy

Atomic Force Microscopy (AFM) was performed with a Dimension Icon (Bruker AXS) instrument in air and under room condition in Tapping mode on a 40 × 40 µm^2^ scan size. High-resolution RTESP (Rotated Tapping Etched Silicon) probes (VEECO Probes, Camarillo, CA, USA) with a sharp tip (radius of curvature specified by the manufacturer R 8 nm) mounted on a rectangular cantilever (length 125 µm, resonant frequency 300 kHz, spring constant 40 N/m) were used for images acquisition. Images were analyzed using the Gwyddion 2.28 free software (http://gwyddion.net/, accessed on 29 December 2021), and the only processing was background subtraction and flattening to remove image slopes and offsets.

### 2.5. Roughness

Roughness Parameters [27] were estimated from the AFM measurements using the software of the own instrument. Amplitude parameters are used to describe characteristics of vertical changes in the surface area, revealing the amplitude of the topography features. The arithmetic average roughness R_a_ is the most widely diffuse descriptor of surface roughness; R_q_ differs from R_a_ in that the deviations of the peak heights and valley depths from the midline are considered as a squared term so that this parameter is believed to be more sensitive to high peaks and deep valleys. R_p_ and R_v_ account for the highest peak and deepest valley in the profile, respectively. Their sum corresponds to R_t_, which thus describes the vertical extent of the profile, from the highest to the lowest points of the profile within the evaluation length. The dimensionless skewness parameter, R_sk_, measures the symmetry of the profile with respect to the mean line. A R_sk_ value of zero pertain surfaces with randomly distributed peaks and valley; the sign of R_sk_ indicates the predominance of peaks (R_sk_ > 0) or valley structures (R_sk_ < 0). The excess kurtosis R_ku_ describes the sharpness of the surface height distribution. A spiky surface will have a high kurtosis value while a gradually varying surface without pronounced peaks or valleys will have a low kurtosis value.

### 2.6. Nanoindentation Measurements

Surface free energy (SFE) measurements were performed using a nanoindentation protocol that was recently presented and validated on a series of reference samples [26]. The method consists of ultra-low load contact experiments to measure the pull-off adhesive forces that are in turn related to the SFE. For these experiments, a sphero-conical diamond tip with a radius (R) of 52.8 µm was adopted. The experiments are performed in pseudo-displacement control mode, with an approaching speed of 10 nm/s. After contacting, the same velocity is maintained until a fixed number of data points, calibrated to ensure that penetration depth into the surface is kept below 10 nm (which roughly corresponds to a maximum load of 15 µN for the flat reference samples), is acquired. In this way, purely elastic deformation of the sample surfaces is ensured and limited, so no damage is caused to the surface. In addition to using an improved actuator, the entire nanoindentation system is installed inside a glove box to control the humidity carefully (RH 8% ± 4%), via a continuous dry nitrogen flow at low inlet pressure (~10 mBar), and temperature (23.0 ± 0.2 °C) during testing. Further details of the employed experimental configuration are reported by Rossi et al. as supplementary information [26]. The measured pull-off forces can be used to calculate the material’s surface free energy (SFE), by using both Johnson–Kendall–Roberts (JKR) and Derjaguin–Muller–Toporov (DMT) models. Both methods were considered for data analysis as DMT is very accurate in describing interactions between stiff bodies where small contact radius are expected while JKR better describes the contact between compliant bodies, where large elastic deformations are prevalent. Thus, the two methods represent the two extremal values of contact–adhesion models for determination of the true value of SFE. The DMT and JKR model are represented by the subsequent equations:

$$\mathrm{SFE}\;\mathrm{DMT}:\;\gamma_{2}={\left(\frac{{-F}_{c}}{4\pi R}\right)}^{2}\cdot \left(\frac{1}{\gamma_{2}}\right)$$

$$\mathrm{SFE}\;\mathrm{JKR}:\;\gamma_{2}={\left(\frac{{-F}_{c}}{3\pi R}\right)}^{2}\cdot \left(\frac{1}{\gamma_{2}}\right)$$

where γ_2_ correspond to the SFE of the sample, R is the tip radius, γ_1_ is the known surface energy of the indenter, and F_c_ is given by the measured pull-off force. Further details of the contact mechanics of these models can be found in [28,29].

Data from at least ten valid measurements for each sample and air-exposure intervals were used. The reference SFE of the diamond indenter tip was taken from the literature, equal to 43 mJ/m^2^ [30].

## 3. Results

### 3.1. Topography Analysis

**Figure 1:**
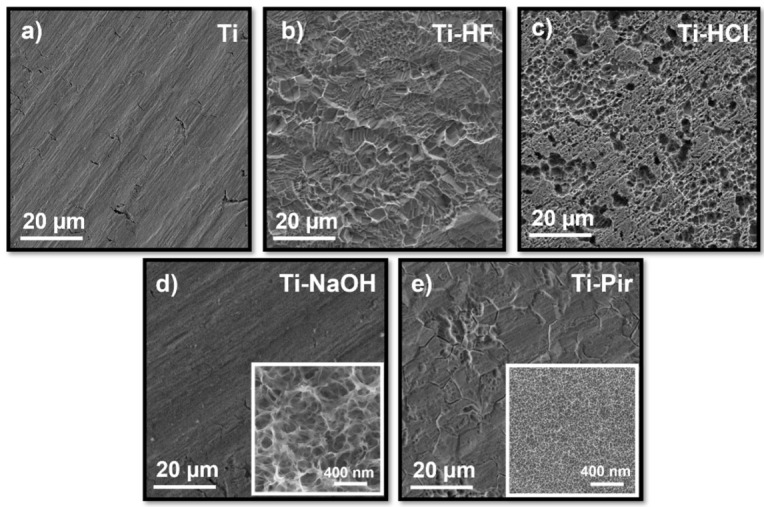
Scanning electron micrographs of untreated commercially pure titanium (**a**), Ti-HF (**b**), Ti-HCl (**c**), Ti-NaOH (**d**), and Ti-Pir (**e**) surfaces. Insets at higher magnification are provided for (**d**,**e**).

Depending on the nature of the etching agent employed, chemical treatments produced quite different surface morphologies, all characterized by an increase in roughness on a multiple scale, as shown in Figure 1. Acid treatments with HCl and HF removed materials from the titanium surface.

In detail, HF produced angular stepped morphology with sharp borders (Figure 1b), while HCl etching primarily generated irregularly distributed deep pits with 1–6 µm diameters (Figure 1c). The typical parallel grooves observed in the non-etched sample (Figure 1a) are still visible in the alkali-treated ones, which showed a smoother and more uniform surface than acid treatments at the same magnification (Figure 1d). A close inspection at higher magnification reveals, for Ti-NaOH, a complex texture consisting of interconnected nanopores (Inset, Figure 1d). Piranha-treated samples show a morphological behavior that is a trade-off between acid and basic treatments. Some similarities with surfaces produced by HF etching are found, as the acidic component involved mostly removed materials along grain boundaries. At higher magnifications, however, the topography recalled the grainy structure produced by the alkali treatment, with a highly dense distribution of nanopores with smaller diameters of 15–30 nm (Figure 1e, inset). Based on the SEM observation, all the etched samples showed a secondary texture at dimensions smaller than the grain structure of titanium, thus exhibiting a complex microporous and nano-rough structure.

**Figure 2:**
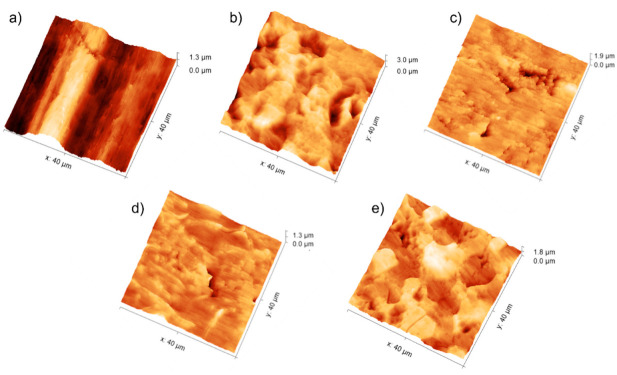
AFM images of the untreated and textured titanium surfaces: Ti (**a**), Ti-HF (**b**), Ti-HCl (**c**), Ti-NaOH (**d**), and Ti-Pir (**e**). The scan size area was fixed to 40 × 40 µm^2^.

Figure 2 shows AFM images of textured titanium specimens recorded keeping the same scale and scan size; the relative roughness parameters are collected in Table 1.

Values obtained for the untreated commercial titanium Ti are strongly influenced by the sample waviness (Figure 2a) that is instead removed by the etching treatments (Figure 2b–e). Subtracting the contribution of waviness to the surface texture, R_a_ for Ti assumes values similar to those typically observed in the literature (Figure S1). Regarding etched surfaces, the evaluation of the amplitude parameters (R_q_, and R_t_) indicate that treated samples are the coarsest followed, in decreasing order of amplitude parameters, by Ti-Pir, Ti-HCl, and Ti-NaOH. Acid pre-treatments produced surfaces with negative skewness (R_sk_ < 0) and positive excess kurtosis (R_ku_ > 0), indicative of a fluctuating morphology with the prevalence of valleys. In contrast, Piranha-treated surfaces showed positive skewness (R_sk_ > 0) and negative excess kurtosis (R_ku_ < 0), resembling a coarse morphology, mainly consisting of islands with different heights. Skewness closed to zero was associated with alkali-etched surfaces, suggesting a symmetrical height distribution and pores with walls of limited height. Quite similar R_w_ values, slightly higher than for untreated Ti, were found for all the etching methods (Table 1) indicating that despite the marked differences in surface morphology, all the treatments produced correspondent real contact area.

### 3.2. Nanoindentation SFE Measurements

**Figure 3:**
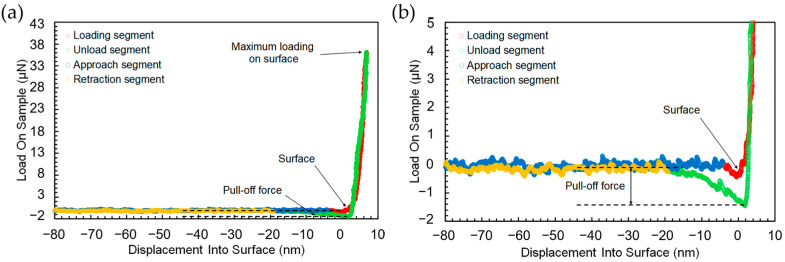
Illustrative SFE nanoindentation measurement response of the reference titanium substrate: (**a**) overall nanoindentation response with interaction segments highlighted, and (**b**) magnification of nanoindentation response to highlight the pull-off adhesion phenomenon.

A representative material response for the applied nanoindentation methodology on untreated titanium substrates is presented in Figure 3. 

**Figure 4:**
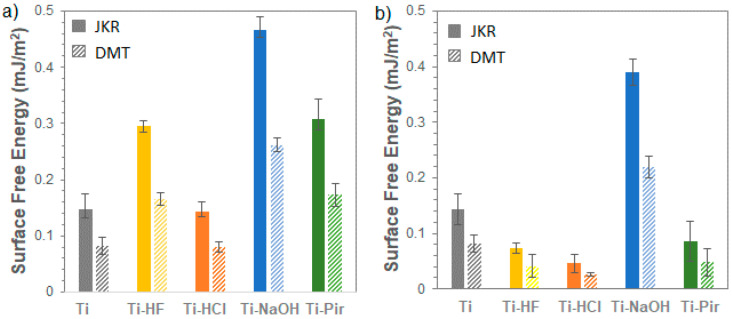
Surface free energy of etched titanium samples (**a**) freshly prepared and (**b**) after 48 h air exposure. Plain bar represents data obtained by JKR model; striped bar data obtained by DMT model.

The relative surface free energy (SFE) values obtained, considering both Johnson–Kendall–Roberts (JKR) and Derjaguin–Muller–Toporov (DMT) models for freshly chemically etched titanium samples in an inert atmosphere and after 48 h in air, are shown in Figure 4. The observed absolute values are significantly lower than those typically derived using the contact angle method (Figure S2).

A first point about this difference can be identified in the intrinsic nature of the nanoindentation method: relying on a purely elastic interaction between a semi-infinite half-space (the sample surface) and a rigid body (indenter tip), the pull-off forces measured by nanoindentation are affected by the sub-micron roughness of the samples, determining the interaction area for the adhesion. This aspect has been discussed in a previous article, where an excellent agreement between the two techniques was obtained in case of atomically flat samples [26].

In this study, however, the changes in roughness and their contribution to SFE were considered as inherent to the surface treatments themselves and thus incorporated in the overall evaluation of the samples’ energy response [31]. The focus of the work is in fact to investigate the time-dependent relative behavior of the different surface treatments comparing samples in equivalent conditions. In this regard, it is worth noting that, when contact angle and nanoindentation measurements can be performed in correspondent conditions, e.g., when the samples are air-stable (Figure 4b), the same relative trends are obtained (Figure S2), and direct comparisons can be made among samples.

Considering data obtained from surfaces measured right after their preparation, a pronounced increase in SFE following treatments with HF, NaOH, and piranha solution is evidenced. Etching with HCl does not appear to have considerably affected the SFE of the Ti substrate, despite the quite different morphology produced (Figure 1). The observed difference in SFE between the HCl and HF acid treatments is mainly due to the different efficiency of removal of the native TiO_2_ layer. As observed from SEM (Figure 1) and AFM (Figure 2) analysis, HF exerts a more incisive action on the substrate, causing crystalline grains from the amorphous titanium structure to detach and produce deep multi-layered conformations. All the natural pre-existing TiO_2_ layer is removed by this treatment, thus exposing the underlying metal characterized by a higher SFE. HCl instead proved to be an excellent decontamination agent in that it could easily dissolve Ti salts that were not weakening Ti surfaces [32], meaning that its etching action has a more limited effect in the removal of the native TiO_2_ layer compared to HF treatment. When it comes to alkali- and piranha-treated surfaces, a different mechanism must be considered. While the TiO_2_ layer dissolves only partially during this type of treatment, the Ti surface is significantly enriched in reactive groups -OH and -O_2_^−^, and -OH, respectively [33]. These highly reactive functionalities account for the observed increase in SFE. It is worth noting here that the nanoindentation-based method we are presenting provides a unique possibility of measuring SFE in a wide range of conditions operating under protective gas. It was possible to measure SFE for highly hydrophilic Ti-NaOH and Ti-Pir surfaces—which both caused liquid drops to completely spread over the surface (Figure S3)—under conditions wholly comparable with those used for all other samples. Assessment and comparison of the actual effect of chemical treatments were then possible, which opens the possibility for a deeper understanding of the observed high reactivity of the treated surfaces. For example, this would be important for the realization of long-term stable active coating and effective functionalization to gain a better knowledge of the mechanism regulating the surface interaction with organic and inorganic molecules.

**Figure 5:**
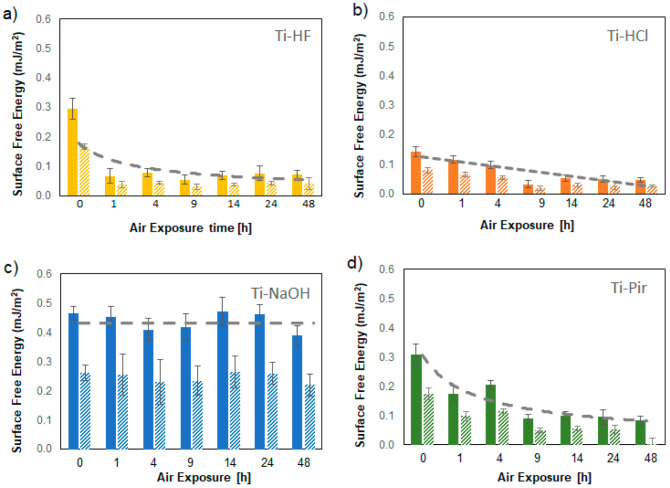
Time-depending SFE of textured titanium surfaces: Ti-HF(**a**), Ti-HCl (**b**), Ti-NaOH (**c**), and Ti-Pir (**d**). Plain bars represent results obtained by applied JKR method; dashed bars are for DMT data.

As observed in the literature, changes in SFE of surfaces upon exposure to air are practically unavoidable and can take place in a short time [31]. SFE values obtained by exposing the chemically treated surfaces to air for controlled times are shown in detail in Figure 5. Thanks to the utterly non-destructive nature of the nanoindentation tests, which do not alter the surface during measurements, it was possible to carry out all the measurements on the same samples of each type. This experimental method thus avoided the problems associated with the intrinsically limited reproducibility of etching procedures. The SFE of the acid-etched surfaces shows a constant decline with aging due to the regrowth of the outer oxide layer and especially to the absorption of carbon-based material due to the contamination of hydrocarbons from the atmosphere. The surface free energy of Ti-HF in particular undergoes an initial rapid drop followed by stabilization, keeping the values reached after just one hour without substantial variation. As reported in the literature [34], the H_2_O_2_ component of the piranha solution was shown to promote the removal from the surface of hydroxyl groups typically associated with the hydrophilic behavior of Ti/TiO_2_ interfaces [35]. This phenomenon, combined with the, although limited, absorption of hydrocarbon, is responsible for the decrease in SFE, which, interestingly, settles at values also observed for the HF-treated surfaces (Figure 3b), in agreement with contact angle suggestion previously collected by our group [36].

Moreover, it is interesting that the decrease in SFE seems to follow a similar relatively slow kinetic for Ti-HCl and Ti-Pir, which are believed to have a limited effect on the native TiO_2_. HF- and Piranha-etched surfaces show almost correspondent initial and final SFE, despite the differences in ageing kinetic, indicating that the acid component probably dominates the SFE effect of the latter treatment. On the contrary, the high surface free energy produced by the alkaline treatment remains constant for the time considered in this study (48 h).

This behavior is likely due to a sodium titanate (Na_2_Ti_5_O_11_) hydrogel-like layer formed during alkali treatment because of the reaction between the dissolved TiO_2_ and OH groups in solution [37]. This protective layer is also responsible for the highly negative character of the surface, accounting for its high reactivity.

Prolonged time measurements of the samples in a protected atmosphere confirmed their excellent stability under these conditions. Apart from Ti-NaOH, all treated surfaces undergo a significant drop in SFE within a few hours of air exposure, reaching a plateau at values lower than those of the untreated reference considered in this work (Figure 4). These observations demonstrate that only alkali treatment can produce highly hydrophilic surfaces unaffected by interaction with the open environment. For all other surface treatments, storage in a nitrogen atmosphere is recommended to preserve the favorable combination of multi-scale roughness and high SFE that has been shown to be necessary to achieve high-performance materials in the biological field [38].

## 4. Conclusions

Our work demonstrated the advantages offered by SFE nanoindentation measurements on surface-treated Titanium samples. This method made it possible to obtain stable and repeatable measurements on the etched surfaces, immediately after their preparation and as a function of time. The same measurements were not accessible by the typical contact angle measurements due to the markedly hydrophilic character initially induced by the treatments with NaOH and piranha solution. Our findings highlighted the importance of a careful assessment of the surface properties at very early stages after chemical treatments to determine the mechanism underlying the transition from a higher SFE, reasonably connected to higher surface reactivity, to quite lower values after a short time of exposure to air, which would affect materials applicability. The described nanoindentation protocol is particularly suitable for the comparative evaluation of evolving situations, as the tip interacting with the surface does not provoke any effect on it, allowing multiple measurements of the same sample under the condition of high reproducibility and reliability. This is not easily obtainable with the usual contact angle measurements due to the pre-wetting effect that one measurement would exert on the next, thus requiring vacuum treatment or the use of multiple replicas. Measurements demonstrated ageing kinetics dependent on the nature of surface etching, suggesting that different mechanisms may contribute to the observed reduction in SFE. Only the alkali treatment generated surfaces with high and stable SFE. All other etchings give surfaces quite sensitive to air exposure that need storage in a protected atmosphere to maintain their initial performance.

## Figures and Tables

**Table 1:** Roughness parameters of the chemically etched titanium surfaces. Measurement errors are within 10% for all parameters and were evaluated considering the results obtained on the same image with different data processing.

Sample	R_a_ (µm)	R_q_ (µm)	R_p_ (µm)	R_v_ (µm)	R_t_ (µm)	R_sk_	R_ku_	R_w_
Ti	0.21	0.26	0.93	0.80	1.73	0.68	−0.29	1.06
Ti-HF	0.33	0.41	1.25	1.75	3.01	−0.48	0.18	1.15
Ti-HCl	0.09	0.12	0.79	0.72	1.51	−0.73	2.72	1.12
Ti-NaOH	0.09	0.11	0.59	0.50	1.09	0.41	0.16	1.11
Ti-Pir	0.20	0.25	0.73	1.07	1.79	0.19	−0.41	1.14

## Data Availability

The data presented in this study are available in this same article and corresponding supplementary materials.

## Supplementary Materials

The following are available online at https://www.mdpi.com/article/10.3390/ma15010287/s1, Figure S1: Resolved contribution of rugosity (green line) and waviness (red line) on the measured texture of non-treated titanium control sample (black line) obtained by Gwyddion Roughness Tool, Figure S2. Owens-Wendt surface free energy of etched titanium samples calculated from static contact angle measurements. Four drops (3 μL) of distilled water and methylene iodide, respectively, were deposited on each surface. Three samples were observed for each condition and the corresponding images were processed by ImageJ software, Figure S3. Images of Ti samples right after the treatment with NaOH (a) and piranha solution (b) showing the complete and disordered spread of H_2_O drops onto the surface.
