# Supplementary material for: A Nanoindentation Approach for Time-Dependent Evaluation of Surface Free Energy in Micro- and Nano-Structured Titanium
Source: Materials (Basel). 2021 Dec 31;15(1):287. doi: 10.3390/ma15010287 (PMC8746133; doi:10.3390/ma15010287)
Supplement: Supplementary file 1 [file materials-15-00287-s001.zip › materials-1512329-supplementary.pdf]

A novel nanoindentation approach for micro- and nanostructured titanium Surface Free Energy determination

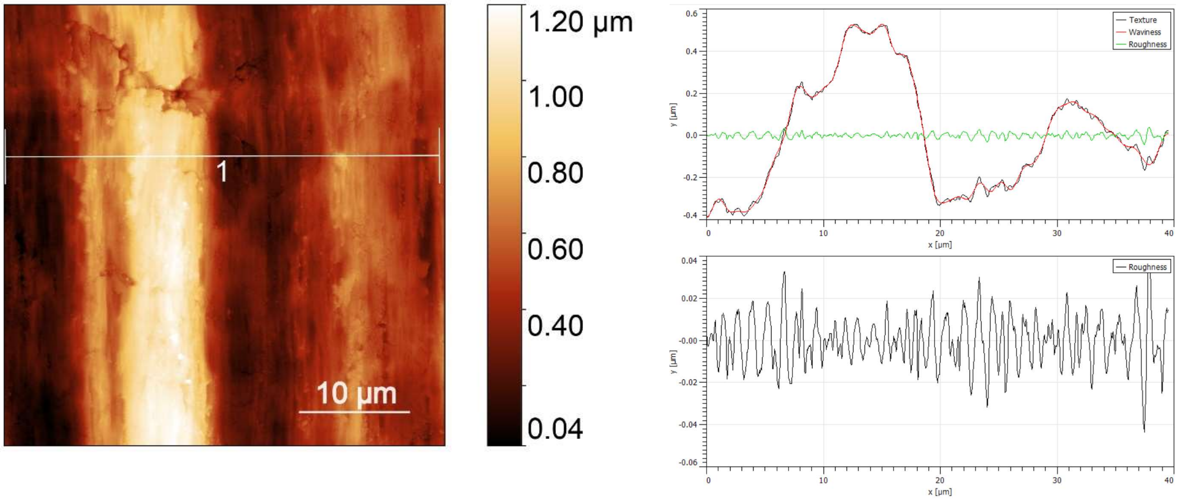

Figure S1. Resolved contribution of rugosity (green line) and waviness (red line) on the measured texture of non-treated titanium control sample (black line) obtained by Gwyddion Roughness Tool.

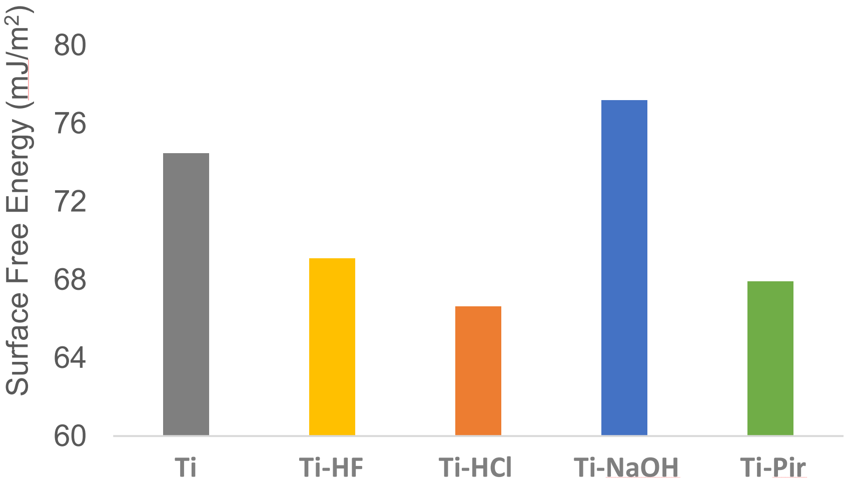

Figure S2. Owens-Wendt Surface free energy of etched titanium samples calculated from static contact angle measurements. Four drops (3 µL) of distilled water and methylene iodide respectively were deposited on each surface. Three samples were observed for each condition and the corresponding images were processed by ImageJ software.

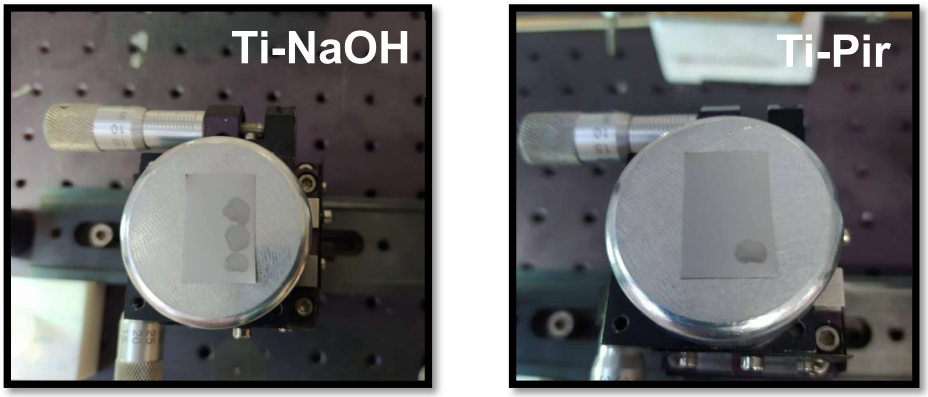

Figure S3. Images of Ti samples right after the treatment with NaOH (a) and piranha solution (b) showing the complete, disordered spread of H_2O drops onto the surface.
